# Guidance for the practical management of the heparin anticoagulants in the treatment of venous thromboembolism

**DOI:** 10.1007/s11239-015-1315-2

**Published:** 2016-01-16

**Authors:** Maureen A. Smythe, Jennifer Priziola, Paul P. Dobesh, Diane Wirth, Adam Cuker, Ann K. Wittkowsky

**Affiliations:** Wayne State University, Detroit, MI USA; Beaumont Health System, Troy, MI USA; University of Nebraska Medical Center College of Pharmacy, Omaha, NE USA; Grady Memorial Hospital, Atlanta, GA USA; Perelman School of Medicine, University of Pennsylvania, Philadelphia, PA USA; University of Washington School of Pharmacy, 1959 NE Pacific St Box 356015, Seattle, WA 98195 USA

**Keywords:** Venous thromboembolism, Anticoagulation, Heparin, Low molecular weight heparin, Enoxaparin, Dalteparin, Fondaparinux, Direct oral anticoagulants (DOAC), New oral anticoagulants (NOAC)

## Abstract

Venous thromboembolism (VTE) is a serious and often fatal medical condition with an increasing incidence. Despite the changing landscape of VTE treatment with the introduction of the new direct oral anticoagulants many uncertainties remain regarding the optimal use of traditional parenteral agents. This manuscript, initiated by the Anticoagulation Forum, provides clinical guidance based on existing guidelines and consensus expert opinion where guidelines are lacking. This specific chapter addresses the practical management of heparins including low molecular weight heparins and fondaparinux. For each anticoagulant a list of the most common practice related questions were created. Each question was addressed using a brief focused literature review followed by a multidisciplinary consensus guidance recommendation. Issues addressed included initial anticoagulant dosing recommendations, recommended baseline laboratory monitoring, managing dose adjustments, evidence to support a relationship between laboratory tests and meaningful clinical outcomes, special patient populations including extremes of weight and renal impairment, duration of necessary parenteral therapy during the transition to oral therapy, candidates for outpatient treatment where appropriate and management of over-anticoagulation and adverse effects including bleeding and heparin induced thrombocytopenia. This article concludes with a concise table of clinical management questions and guidance recommendations to provide a quick reference for the practical management of heparin, low molecular weight heparin and fondaparinux.

## Introduction

Heparin has been a component of the initial treatment of venous thromboembolism (VTE) for decades. Despite its long history, various aspects of the practical use of unfractionated heparin (UFH), whether delivered intravenously (IV) or subcutaneously (SC), continue to challenge clinicians. In 1998, the US FDA approved the low molecular weight heparin (LWMH) enoxaparin (Lovenox) for VTE treatment, followed by approval of the synthetic heparin-like compound fondaparinux (Arixtra) in 2004. In 2007, the LMWH dalteparin (Fragmin) was approved for VTE treatment in patients with cancer. These agents, intended for subcutaneous administration, offer practical advantages over unfractionated heparin, yet present their own challenges, particularly in special populations. This chapter will address the practical use and management of the parenteral heparin anticoagulants available in the US when used in the treatment of VTE.

## Methods

To provide guidance on the practical management of the heparin anticoagulants in adults, we first developed a number of pivotal practical questions pertaining to each of the commonly used heparin compounds (UFH, LMWH and fondaparinux) to be reviewed in this document (Table 1). Questions were developed by consensus from the authors. The literature addressing the above questions was reviewed by searching electronic databases (PubMed, Medline) and the authors’ personal libraries, with a focus on high quality cohort studies and randomized controlled trials published in the last 10 years, where available. For each question, a brief summary and interpretation of pertinent literature and existing guidelines, where available, are provided, followed by guidance to the reader.

**Table 1:** Guidance questions to be considered

Heparin for treatment of acute VTE
(1) How should heparin be initiated, including baseline laboratory tests and dosing?
(2) What weight should be used to calculate dosing, and should obese and low body weight patients be treated differently?
(3) How should heparin be monitored?
(4) What data support the benefit of monitoring?
(5) What is the appropriate therapeutic range?
(6) When should heparin resistance be suspected?
(7) What algorithm should be used for dosing adjustments?
(8) What is the appropriate duration of therapy for heparin for transition to oral anticoagulant therapy?
(9) How should heparin-induced over-anticoagulation, thrombocytopenia and bleeding be managed?
LMWH for treatment of acute VTE
(1) How should LMWH be initiated, including baseline laboratory tests and dosing?
(2) What weight should be used to calculate dosing, and should obese and low body weight patients be treated differently?
(3) How should patients with renal impairment be treated?
(4) How should routine treatment be monitored?
(5) Is there a role for peak anti-Xa monitoring and for trough anti-Xa monitoring?
(6) What is the appropriate duration of therapy when transitioning to oral anticoagulant therapy?
(7) Which patients are acceptable candidates for outpatient treatment of VTE with LMWH?
(8) How should LMWH-induced over-anticoagulation, thrombocytopenia and bleeding be managed?
Fondaparinux for treatment of acute VTE
(1) How should fondaparinux be initiated, including baseline labs and dosing?
(2) What weight should be used to calculate dosing, and should obese and low body weight patients be treated differently?
(3) How should patients with renal impairment be treated?
(4) How should treatment be monitored?
(5) Is there a role for peak anti-Xa monitoring and for trough anti-Xa monitoring?
(6) What is the appropriate duration of therapy when transitioning to oral anticoagulant therapy?
(7) Who is a candidate for outpatient treatment of VTE with fondaparinux?
(8) Can fondaparinux be used for VTE treatment in the presence of active heparin-induced thrombocytopenia (HIT) or those with a history of HIT?
(9) How should fondaparinux-induced over-anticoagulation and bleeding be managed?

## Guidance

### Heparin for the treatment of acute VTE

1. How should heparin be initiated, including baseline laboratory tests and dosing?

While heparin therapy for VTE treatment is typically administered by continuous IV infusion, both adjusted dose and fixed dose SC injections can also be utilized. A comparative study of SC and IV heparin using the same initial dose (5000 unit IV bolus followed by 30,000 units/day) reported an increased risk of VTE recurrence with SC heparin (19.3 % vs. 5.2 %; *p* = 0.024), suggesting the need for higher doses with this route [1]. A meta-analysis comparing IV heparin to dose-adjusted SC heparin (initial dose 5000 units IV then 17,500 units SC twice daily) for initial DVT treatment found a lower risk of VTE recurrence or extension with SC heparin (relative risk [RR] 0.62, 95 % confidence interval [CI] 0.39–0.98) with a similar risk of major bleeding (RR 0.79, 95 % CI 0.42–1.48) [2]. Prandoni et al. conducted an open label multicenter trial in 720 patients comparing LMWH to adjusted dose SC heparin in those with acute, symptomatic VTE. SC heparin regimens were weight based with those over 70 kg receiving an initial 6000 unit IV bolus then 17,500 units twice daily. Objectively confirmed recurrent VTE, major bleeding and overall mortality were similar [3]. In the fixed dose heparin investigators (FIDO) trial, a randomized, open label, non-inferiority trial, fixed dose heparin (333 units/kg bolus then 250 units/kg every 12 h) was compared to LWMH for initial treatment of acute VTE [4]. There was no weight exclusion for the trial. Recurrent VTE at 3 months occurred in 3.8 % of heparin patients compared to 3.4 % of LMWH patients (absolute difference 0.4 %; 95 % CI −2.6 to 3.3 %) while major bleeding occurred in 1.1 % of heparin patients versus 1.4 % of LMWH patients (absolute difference, −0.3 %; 95 % confidence interval, −2.3 to 1.7 %).

The optimal initial dosing of continuous infusion heparin therapy is controversial. In 1989, the second American College of Chest Physicians (ACCP) Clinical Practice Guideline on VTE treatment recommended an initial 5000 unit bolus followed by a 1000 unit/h infusion [5]. In 1993, Raschke et al. compared weight based heparin dosing (80 units/kg followed by 18 units/kg/h) to a standard regimen (5000 units followed by 1000 units/h) in 115 patients with venous or arterial thrombosis [6]. A five-fold reduction in recurrent VTE was observed with weight-based dosing (95 % CI 1.1–21.9). Nevertheless, most VTE treatment trials incorporated a fixed dose initial heparin infusion regimen of 5000 unit bolus followed by infusion of approximately 1300 units/h [7]. In 1992, the ACCP VTE treatment guidelines suggested a 5000–10,000 unit bolus followed by a fixed heparin infusion of 1300 units/h (31,200 units/day) and in 1995 and 2004 they endorsed either a fixed regimen or the Raschke weight based regimen [8–10]. For a 70 kg patient, the Raschke regimen translates into a heparin bolus of 5600 units followed by infusion of 1260 units/h. The 2012 version of the guidelines do not address UFH dosing in the VTE treatment chapter [11]. However, in the chapter on parenteral anticoagulants, UFH dosing recommendations are similar to those in 1995 and include either a weight based regimen (Raschke regimen) or a fixed regimen of 5000 unit bolus followed by a continuous infusion of at least 32,000 units/day [12].

The Joint Commission’s National Patient Safety Goals require that a written policy stating required baseline and ongoing laboratory tests for patients on heparin be in place in healthcare institutions [13]. Pre-treatment hemoglobin and hematocrit are used as a baseline from which to assess subsequent changes that may reflect bleeding. A baseline platelet count is used to compare to subsequent values in order to detect the possible development of heparin-induced thrombocytopenia (HIT). Finally, an elevated pre-treatment prothrombin time (PT) or activated partial thromboplastin time (aPTT) may detect the presence of an underlying coagulation defect.

#### **Guidance Statement**

*We suggest that total body weight, CBC, PT and aPTT be obtained prior to initiating heparin therapy. Heparin efficacy is related to dose regardless of route. The initial dose is more important than the aPTT in predicting efficacy. Although optimal initial dosing for bolus and continuous infusion remain uncertain, we suggest doses outlined in Table* *2**, acknowledging that these options have not been compared in head*-*to*-*head clinical trials. Internal audits to determine the dose requirement to produce therapeutic anticoagulation based upon the responsiveness of the health*-*system’s aPTT reagent and coagulation instrument are strongly encouraged.*

**Table 2:** Recommended initial dosing for UFH in VTE treatment

Route	Reference	Bolus dose	Maintenance dose
Continuous infusion			
Fixed dose	Hull et al. [1]	5000 units	1250–1280 units/h^a^
Weight based	Raschke et al. [6]	80 units/kg	18 units/kg/h
Subcutaneous			
Fixed dose	Kearon et al. [4]	333 units/kg	250 units/kg every 12 h
Adjusted dose	Prandoni et al. [3]	5000 units	17,500 units every 12 h adjusted to aPTT

^a^Caution is needed with low body weight patients; individualized dosing should be considered

(2) What weight should be used to calculate dosing and should obese and low body weight patients be treated differently?

The volume of distribution of heparin approximates blood volume, is related to body weight and averages 60 mL/kg [14]. There are no prospective trials evaluating heparin dosing regimens using different weight strategies, although a trial in obese patients (NCT01361193) is ongoing. Current dosing recommendations do not specify which weight should be used. The Raschke nomogram used actual body weight; however only 9/115 patients (<8 %) had a weight above 100 kg (range 101–131 kg) [6].

For the obese and morbidly obese patient, the importance of striking a balance between achieving effective anticoagulation and avoidance of bleeding is apparent. Although obese patients have a larger blood volume, the vascularity of adipose tissue is lower than that of lean body mass, raising concern for over-anticoagulation when heparin is dosed using total body weight. Under-dosing is also a concern as obese patients are at increased risk of VTE recurrence. Dose caps employed to increase safety may increase the risk of under-dosing in the obese/morbidly obese patient and contribute to treatment failures.

Heparin dosing in obesity/morbid obesity has recently been reviewed [15]. Current data are limited by the low quality of evidence (case report, case series, retrospective reports), the lack of a standard definition of obesity and the small number of patients evaluated. Patient management strategies include dosing based on total body weight, ideal body weight, adjusted body weight, or total body weight with a reduced infusion rate. Protocols based on total body weight increase the risk of a supra-therapeutic aPTT; however no increase in bleeding has been reported [16]. Heparin infusion rates resulting in therapeutic anticoagulation have ranged from 5 to 12.8 units/kg/h in the obese/morbidly obese population [14]. Definitive conclusions as to which weight should be used cannot be drawn due to the low quality of evidence in this area. As for underweight patients, there are no data evaluating the most appropriate heparin dosing but such patients may be at increased bleeding risk [17].

#### **Guidance Statement**

*When a weight based heparin dosing strategy is selected, we suggest total body weight for calculating dose. For the obese/morbidly obese patient either total body weight or adjusted body weight can be used. Although no increased risk of major bleeding has been reported when morbidly obese patients are managed using total body weight, studies have not included patients weighing above 270* *kg. If adjusted body weight is used, prompt attention to initial laboratory results is warranted to ensure the therapeutic threshold is exceeded in a timely manner. Empiric dose caps may increase the risk of initial under anticoagulation in obese and morbidly obese patients. If empiric dose caps are used, individualized initial dosing should be available for obese and morbidly obese patients.*

(3) How should heparin be monitored?

The aPTT, which measures the function of the intrinsic and common clotting pathways, is the most commonly used laboratory test to monitor heparin. Numerous variables impact the aPTT result including pre-analytic (sample collection and processing), analytic (reagent and instrument) and biologic factors (level of clotting factors) [18]. Over 300 different reagent-instrument combinations are used clinically. A therapeutic heparin level (0.3–0.7 u/mL) by anti-factor Xa (anti-Xa) analysis can produce aPTT ratios ranging from 1.6–2.7 to 3.7–6.2 times control depending upon the reagent/coagulometer combination [11]. In response to the numerous limitations of the aPTT, researchers have evaluated direct heparin concentration monitoring using heparin anti-Xa levels. Compared to the aPTT, the heparin anti-Xa level is less impacted by biologic variables, but pre-analytic and analytic variability remain and can be considerable [19]. The anti-Xa is also more expensive than the aPTT, and is both less available and less familiar to clinicians.

A recent review identified the potential advantages of the heparin anti-Xa level over the aPTT for heparin monitoring. Advantages included fewer monitoring tests, fewer dose changes and a shorter time to obtain therapeutic anticoagulation [20]. Large VTE trials evaluating patient outcomes with heparin anti-Xa level monitoring are not available. Although both the aPTT and the anti-Xa level can be used to monitor heparin, paired results within individual patients are often discordant [21]. In a recent trial in which clinical outcomes of aPTT versus anti-Xa monitoring were evaluated, a disproportionate prolongation of the aPTT relative to the anti-Xa level was the most common discordant pattern [22]. Patients with relatively high aPTT to anti-Xa levels had higher rates of major bleeding and death compared to patients with concordant paired test results. National guidelines for heparin monitoring recognize the limitations of both approaches without recommending a preferred approach [23].

Direct anti-Xa level monitoring is recommended in those with heparin resistance (see subsequent section), baseline aPTT elevation from a lupus anticoagulant or contact factor deficiency or those with markedly elevated levels of fibrinogen or factor VIII [24].

#### **Guidance Statement**

*The optimal approach to heparin monitoring is unknown. Either aPTT or heparin anti*-*Xa level monitoring may be used. We suggest using anti*-*Xa level monitoring in patients with heparin resistance, a prolonged baseline aPTT or altered heparin responsiveness. We suggest the aPTT or anti*-*Xa level be checked every 6* *h until two consecutive therapeutic results are obtained, after which the frequency of monitoring can be extended to once daily.*

(4) What data support the benefit of monitoring?

*Monitoring aPTT*

Despite the standard practice to monitor heparin through coagulation laboratory testing, the body of evidence supporting monitoring is surprisingly weak. In 1972, Basu et al. reported a lower rate of VTE recurrence when the aPTT was maintained between 1.5–2.5 × control [25]. A subsequent study using a rabbit model of thrombosis demonstrated prevention of thrombus extension with an aPTT of approximately 1.5 times control [26]. These data provided the foundation for an empiric aPTT therapeutic range of 1.5–2.5 times control. Over the next two decades, important concepts emerged, which challenged the accepted aPTT therapeutic range as well as the overall benefit of monitoring heparin therapy. Data supporting a relationship between sub-therapeutic aPTTs within the first 24–48 h and increased VTE recurrence at 90 days came from post hoc analysis of studies using fixed dose heparin regimens of ≤30,000 units/day [7]. A pooled analysis of trials using initial heparin infusion regimens of at least 30,000 units/day showed no association between sub-therapeutic aPTT and increased VTE recurrence (90 day recurrence: 6.3 % with sub-therapeutic aPTT in first 24–48 h versus 7 % in those with therapeutic aPTTs; odds ratio 0.89, 95 % CI: 0.2–4) [27]. In the FIDO trial of unmonitored SC heparin, recurrent VTE (at 90 days) occurred in 3.8 % of heparin patients versus 3.4 % with LMWH, absolute risk difference 0.4 (95 % CI −2.6 to 3.3) [4]. Although therapy was unmonitored, on day 3 an aPTT was drawn and subsequently analyzed by a central laboratory at study conclusion. None of the heparin patients with recurrent VTE had a sub-therapeutic aPTT [4]. Data supporting the upper limit of the heparin aPTT therapeutic range for VTE are even weaker than data supporting the lower limit [7, 27–30]. Hull et al. reported bleeding events in 8.6 % of VTE patients with supratherapeutic aPTTs compared to 12.3 % of patients without [28]. In the FIDO trial none of the major bleeding events in the heparin group were associated with an aPTT above 85 s [4].

*Monitoring heparin levels*

Protamine sulfate titration and anti-Xa analysis are two approaches to heparin level monitoring. Early animal studies suggested a heparin level therapeutic range of 0.2–0.4 units/mL by protamine sulfate titration [18]. Subsequent studies from McMaster University demonstrated that this range was equivalent to a heparin anti-Xa level of approximately 0.3–0.7 units/mL [31]. This equivalency between assay systems was promoted by national guidelines despite data challenges to its validity [12, 24]. Since appearing in national guidelines, the heparin anti-Xa level therapeutic range of 0.3–0.7 unit/mL has gained widespread acceptance despite limited clinical trial outcome data [31]. In 2008, the ACCP VTE treatment guidelines stated “When patients are treated with an initial heparin infusion of 1250 U/h (corresponding to 30,000 U/d) or 18 units/kg/hr, it is uncertain if adjustment of heparin dose in response to the aPTT or heparin levels improves efficacy or safety [32]. There are no recent trials evaluating unmonitored continuous infusion heparin therapy or different levels of heparin anticoagulation.

#### **Guidance Statement**

*The benefit of monitoring IV heparin once a therapeutic threshold has been exceeded is not well defined. We suggest monitoring of continuous infusion heparin therapy, either using aPTT or anti*-*Xa, as this is considered standard of care despite the weak evidence base. Monitoring is optional in those receiving SC weight*-*based heparin therapy.*

(5) What is the appropriate therapeutic range?

The Raschke study, the pivotal trial for weight based IV heparin dosing, used a fixed therapeutic aPTT interval of 46–79 s which corresponded to an aPTT ratio of 1.5–2.3 times control [6]. Other VTE treatment trials comparing heparin to LMWH employed a fixed aPTT ratio of 1.5–2.5 times control for heparin patients. In recognition of the variable sensitivity of different aPTT reagents to heparin, the use of an empiric fixed interval or fixed aPTT ratio for heparin monitoring is no longer recommended for treatment of VTE in national guidelines. The College of American Pathologists (CAP) and the ACCP recommend that each institution define its own therapeutic aPTT range based upon the responsiveness of the aPTT reagent and coagulometer in use [12, 23, 24]. The therapeutic range should be re-established with each change of reagent manufacturer, lot or coagulation instrument. Ex-vivo samples (as opposed to spiked samples) from 30 patients receiving therapeutic dose heparin should be used. A 3.2 % sodium citrate concentration is recommended for sampling. Two approaches are acceptable according to the CAP. The first is to use linear regression analysis to determine the aPTT interval which corresponds to a therapeutic heparin concentration (e.g. 0.3–0.7 anti-Xa units/mL). Although this approach was previously recommended by the ACCP, the most recent guidelines do not specify a desired therapeutic range for use of heparin in the treatment of VTE [11, 12, 32]. The use of a heparin concentration-calibrated aPTT therapeutic range for heparin monitoring has not been prospectively evaluated against alternative monitoring strategies.

The second approach recommended by the CAP compares aPTT results using the old and new reagent and assesses reagent drift using the cumulative summation method. Differences in aPTT results are tracked yearly and adjustments in therapeutic range are needed when the cumulative difference exceeds 7 s [24]. The CAP recommends the first approach to establish the initial heparin therapeutic range and the cumulative summation method for subsequent changes in reagent. If direct heparin concentration monitoring is used, a therapeutic range of 0.3–0.7 U/mL by anti-Xa analysis is widely accepted and promoted despite limited data [18, 24, 31, 33].

#### **Guidance Statement**

*The optimal heparin therapeutic range is uncertain. The target therapeutic range is less important than ensuring an appropriate initial heparin dose. The CAP suggests the one*-*time establishment of a heparin concentration*-*derived aPTT therapeutic range. The cumulative summation method is suggested for range re*-*evaluation following reagent/instrument change. When anti*-*Xa monitoring is used, a therapeutic target of 0.3–0.7 units/mL is suggested.*

(6) When should heparin resistance be suspected?

Heparin resistance is a term used to describe patients who require high doses of heparin. Heparin resistance can be caused by antithrombin deficiency, increased heparin clearance or increased heparin binding proteins (often considered acute phase reactants). Patients with these conditions have a reduction in the formation of heparin-antithrombin complexes and require higher heparin doses to reach a therapeutic aPTT. Although the term heparin resistance is also used to describe patients with increased levels of factor VIII or fibrinogen, these individuals may more appropriately be described as having altered heparin responsiveness. These patients have a downward shift in the dose response curve resulting in a shortened or blunted aPTT response. Such patients may appear to be heparin resistant but increasing the heparin dose based upon the aPTT may lead to over-anticoagulation and an increased risk of bleeding [34].

Heparin resistance is commonly defined as a daily dose in excess of 35,000 units/day [31, 33]. This may be an inappropriately low threshold, as a dose of 19 units/kg/h for an 80 kg patient would exceed this daily dose. A weight based definition of resistance (units/kg/h) may be more appropriate but consensus is lacking. In a study of “heparin resistant” (requiring >35,000 units/day) patients, Levine et al. compared anti-Xa monitoring to aPTT monitoring. Patients randomized to aPTT monitoring required higher heparin doses while those randomized to anti-Xa level monitoring had subtherapeutic aPTTs during the majority of treatment. Rates of major bleeding and thrombosis were similar [35].

#### **Guidance Statement**

*We suggest drawing a paired aPTT and heparin anti*-*Xa level when heparin resistance is suspected. If the aPTT is subtherapeutic and the anti*-*Xa level is therapeutic, the heparin dose does not require adjustment and subsequent monitoring should occur using the anti*-*Xa level when feasible. If, despite serial dose increases, both the aPTT and anti*-*Xa level remain low, true heparin resistance may be present.*

(7) What algorithm should be used for dosing adjustments?

The use of a nomogram to guide heparin dosage adjustment increases the proportion of patients receiving adequate anticoagulation based on achieving a therapeutic aPTT [35]. Even with nomograms, however, nontherapeutic aPTTs occur in more than 25 % of patients in clinical trials [36]. In one study, only 29 % of patients with a therapeutic aPTT had two consecutive repeat therapeutic aPTTs [37]. Dose adjustment algorithms can be weight based (e.g. increase infusion rate by 2 units/kg/h) or fixed dose (e.g. increase infusion rate by 100 units/h) [6, 38]. Dose adjustment algorithms were not provided in the major VTE treatment trials comparing heparin to LMWH. A fixed dose algorithm may be inadequate in obese patients [15]. In a single center study, Cruickshank et al. evaluated the performance of a dosage adjustment nomogram by calculating the success rate for each heparin dose adjustment recommendation based upon the aPTT interval (e.g. increase heparin infusion by 80 units/h for an aPTT of 50–59 s) [38]. Results were used to modify the dosage adjustment algorithm. No studies are available which compare different heparin dosage adjustment algorithms. Health-systems typically adopt a published algorithm from a single center trial [6, 39].

#### **Guidance Statement**

*We suggest that heparin dosing be guided by a dose adjustment nomogram, and that a weight based heparin dose adjustment algorithm may offer benefit over a fixed adjustment algorithm for the obese patient. More research in defining and assessing the optimal dosage adjustment nomogram is needed.*

(8) What is the appropriate duration of therapy for transition to oral anticoagulant therapy?

Patients with VTE who are treated with a vitamin K antagonist alone have an unacceptably high rate of VTE extension and recurrence [40]. The ACCP recommendations for the desired duration of overlap of a parenteral agent with warfarin over the past 15 years are summarized in Table 3 [10, 11, 32, 41]. The current recommendation is to continue parenteral anticoagulation along with warfarin for a minimum of 5 days and until the INR is 2.0 or above for at least 24 h [11]. The 5 day minimum is recommended to avoid a potential hypercoagulable state upon warfarin initiation. Factor II levels take a minimum of 5 days to fall while protein C (an endogenous anticoagulant) has a short half-life and is more rapidly depleted [42]. The recommendation for an INR of 2 or greater for at least 24 h is provided to ensure that the INR elevation indicates adequate anticoagulation and not solely a reduction in factor VII (short half-life). The ACCP recommended duration of overlap was followed in some VTE treatment trials demonstrating similar efficacy of LMWH to heparin (new VTE at 90 days in heparin patients 1.9 and 4.1 %) while others included a 6 day overlap with a single INR requirement above 2.0 (recurrent 90 day VTE in heparin patients 6.8 and 6.9 %) [28, 43–45]. In a retrospective study conducted in an academic medical center, Hylek et al. reported compliance with the ACCP recommendation for overlap in only 20 % of patients with more than 40 % of surgical patients having less than a 4 day overlap [37]. For hospitalized patients this recommendation is often perceived to increase length of stay. Patients may be discharged on LMWH to complete the requisite period of overlap, but this temporary measure complicates the transition of care.

**Table 3:** History of ACCP recommendations for overlapping parenteral anticoagulants with warfarin for the treatment of VTE

Year	VTE recommendation	Level of evidence
2001 [41]	Treat with heparin or LMWH for at least 5 days and *overlap with heparin or LMWH for at least 4*–*5* *days*	1A (in comparison to treatment for 10 days)
2004 [10]	Initiate vitamin K antagonist with LMWH or heparin on day one and *discontinue heparin when INR is stable and* >*2.0*	1A
2008 [32]	Treat with LMWH, heparin or fondaparinux for *at least 5* *days and until the INR is* ≥*2 for 24* *h*	1C
2012 [11]	Recommend early initiation of vitamin K antagonist (same day as parenteral is started) over delayed initiation, and *until the INR is 2.0 or above for at least 24* *h*	1B

It is important to note that the ACCP recommendation for an INR > 2 for at least 24 h is paired with the recommendation to treat with a parenteral agent for at least 5 days (a separate level of evidence does not exist for the INR recommendation). Recently, there is noticeable movement away from requiring the INR to be above 2 *for at least 24* *h*. The 2015 Joint Commission VTE Core Measures (VTE-3) address anticoagulant overlap requirements [46]. Compliance requires a minimum of 5 days of overlap with a heparin product and warfarin (or discharge on both agents or documentation of why overlap is not indicated) and a single INR above 2 prior to discontinuation of parenteral therapy. This approach has been used in recent clinical trials [47].

Dabigatran, edoxaban, rivaroxaban and apixaban are FDA approved for the treatment of VTE. These target-specific oral anticoagulants (TSOACs) achieve their anticoagulant effect within 2–3 h of oral administration [48]. Nevertheless, when dabigatran or edoxaban are used for VTE treatment, they must be started after a minimum of 5 days of parenteral anticoagulant therapy, according to manufacturer recommendations based on study design [47, 49, 50].

The direct acting Xa inhibitors rivaroxaban and apixaban do not require initial injectable therapy, but many patients in the clinical trials did receive an initial dose of a parenteral anticoagulant [51–53].

Recommendations for transitioning from parenteral to oral anticoagulants are described in Table 4.

**Table 4:** Transitions from parenteral to oral anticoagulants in the treatment of VTE

	To warfarin	To dabigatran or edoxaban	To rivaroxaban or apixaban
Initial parenteral therapy	Required	Required	Not required
From heparin	Start warfarin and heparin concurrently	Start heparin alone	Stop heparin
	Continue heparin for a minimum of 5 days AND until INR > 2.0	After a minimum of 5 days of heparin, start dabigatran or edoxaban and stop heparin	Give first dose of rivaroxaban or apixaban
From LMWH or fondaparinux	Start warfarin and LMWH/fondaparinux concurrently	Start LMWH/fondaparinux alone	Stop LMWH/fondaparinux
		After a minimum of 5 days, stop LMWH/fondaparinux	
	Continue LMWH/fondaparinux for a minimum of 5 days AND until INR > 2.0	Give first dose of dabigatran or edoxaban at the time the next dose of LMWH/fondaparinux would have been given	Give first dose of rivaroxaban or apixaban at the time the next dose of LMWH/fondaparinux would have been given

#### **Guidance Statement**

*Parenteral anticoagulation with heparin should be overlapped with warfarin for at least 5* *days and until a single INR is 2.0 or greater. Treatment of VTE with rivaroxaban and apixaban does not require initial parenteral anticoagulation while dabigatran and edoxaban require a minimum of 5* *days of parenteral anticoagulation prior to initiation. See Table* *4**for additional details.*

(9) How should heparin-induced over-anticoagulation, thrombocytopenia and bleeding be managed?

The rate of heparin-associated major bleeding is 3 % in recent VTE treatment trials and increases to 4.8 % in a real world practice setting [37, 54]. Failure to follow a dosage adjustment algorithm may increase bleeding risk. When major bleeding occurs and reversal of heparin’s effect is desired, protamine sulfate can be administered. A dose of 1 mg of protamine per 100 units of heparin is recommended. Because of its relatively short half–life, only heparin administered over the past few hours should be considered in calculating the dose of protamine [54]. In emergent situations, clinicians can typically administer 25 mg of protamine for those on continuous infusion therapy with repeat dosing if needed. Protamine has its own significant side effects including allergic reactions, hypotension, bradycardia and respiratory toxicity. Protamine must be given by slow IV infusion at doses ≤5 mg/min.

HIT is a paradoxical adverse effect of heparin which can result in life threatening thrombosis. HIT is suspected more often than the diagnosis is confirmed and over-treatment is a growing concern. Once suspected, a 4 T score (Timing, Thrombocytopenia, Thrombosis, Other) should be calculated to evaluate the likelihood of HIT [55]. In those with a moderate to high 4 T score (scores of 4–8) serologic testing should be performed, all heparin products must be discontinued and alternative anticoagulant therapy should be initiated. If the diagnosis is confirmed with appropriate serologic testing, HIT should be added to the problem list and heparin to the allergy list in the patient’s electronic health records and the patient should be educated to avoid heparin. Patients with HIT-associated thrombosis require anticoagulation therapy, typically for a period of 3–6 months. Warfarin should not be initiated until platelet count recovery and should be overlapped with a parenteral anticoagulant until the INR reaches the therapeutic range. The reader is referred to a recent review on HIT for more details on patient management [55]. Development and implementation of HIT guidelines may improve the outcomes of those with HIT, reduce unnecessary alternative anticoagulant use, decrease cost and improve anticoagulant safety [56].

#### **Guidance Statement**

*We suggest protamine sulfate be administered to reverse the effect of heparin when indicated. We suggest that health systems develop and implement guidelines on anticoagulant reversal and HIT evaluation and management.*

## Guidance

### LMWH for the treatment of acute VTE

1. How should LMWH be initiated, including baseline laboratory tests and dosing?

Enoxaparin (Lovenox) and dalteparin (Fragmin) are the LMWHs available in the US. Enoxaparin is approved for inpatient treatment of acute DVT with or without PE and for outpatient treatment of acute DVT without PE using 1 mg/kg SQ q12 h or 1.5 mg/kg q24 h [57]. Dalteparin is approved for VTE in patients with cancer at a dose of 200 units/kg SQ q24 h [58]. Dosing is generally based on total body weight and renal function, evaluated using the Cockcroft-Gault method, further influences dosing requirement [59].

Use of enoxaparin 1.5 mg/kg once daily for the treatment of VTE is controversial. This dosing option is based on a single randomized, clinical trial comparing unfractionated heparin (UFH) to enoxaparin 1.5 mg/kg daily or 1 mg/kg twice daily in 900 patients with VTE [43]. While there was no difference in recurrent VTE or major bleeding between the groups as a whole, only 32 % of the patients enrolled had PE at the time of randomization. Patients with symptomatic PE, obesity and malignancy all had higher rates of recurrent VTE when treated with 1.5 mg/kg daily versus 1 mg/kg twice daily. Current guidelines suggest that when enoxaparin is used for the treatment of VTE, it should be dosed at 1 mg/kg twice daily and that the reduced dose delivered by 1.5 mg/kg once daily be avoided [11, 60].

Limiting dalteparin to the treatment of cancer-associated VTE is not necessary. Dalteparin is highly effective for the treatment of VTE in patients without malignancy using 200 units/kg once daily or 100 units/kg twice daily as determined in a number of clinical trials [11, 61, 62].

Although the risk of HIT with LMWH is lower than with UFH, a baseline platelet count is recommended as a basis from which to consider the development of HIT. Re-exposure to LMWH should be avoided in a patient with a known history of HIT [63].

Therapy should be initiated as soon as possible, as long as it is determined that fibrinolytics are not going to be administered for acute VTE. A pre-treatment hemoglobin and/or hematocrit are used as a baseline from which to assess subsequent changes that may reflect bleeding. Finally, an elevated pre-treatment PT or aPTT may detect the presence of an underlying coagulation defect.

#### **Guidance Statement**

*We suggest that total body weight, baseline serum creatinine, CBC, PT and aPTT be obtained prior to initiating LMWH. We suggest that when enoxaparin is used for the treatment of VTE, only the twice daily dosing strategy be used, except in patients with severe renal insufficiency (see below). Further, we suggest that once daily dalteparin can be used for the treatment of both cancer*- *and non*-*cancer*-*associated VTE.*

(2) What weight should be used to calculate dosing, and should obese and low body weight patients be treated differently?

In clinical trials evaluating the effectiveness and safety of LMWH for the treatment of VTE, total body weight has been used to calculate dosing. While data evaluating the safety and efficacy of LMWH in VTE patients with extremes of weight is limited, total body weight is recommended for LMWH dosing [59]. Due to concerns that dosing based on total body weight may lead to over-anticoagulation in obesity, dose capping has been suggested and is recommended in the product information for dalteparin. However, several studies show little to no accumulation in patients given uncapped doses with body weights up to 190 kg with dalteparin and 159 kg with enoxaparin [59, 64]. In addition, limiting the dose of LMWH by using dose capping may result in inadequate anticoagulation and an increased risk of recurrent VTE [65].

Pharmacodynamic studies involving LMWHs have included patients weighing up to 190 kg, and the maximum weight of patients enrolled in clinical trials is 196 kg [64, 66]. In a retrospective cohort of 300 patients receiving enoxaparin for VTE treatment, the incidence of bleeding events was similar between patients with a BMI ≥ 40 kg/m2; (maximum 66.4 kg/m^2^) and non-obese patients (29 % vs. 23.1 %, *p* = 0.43) [67]. A multivariate analysis concluded that obesity was not associated with an increased risk of bleeding. The average dose of enoxaparin was clinically similar between the groups (0.98 mg/kg vs. 1.04 mg/kg) with the majority of patients receiving twice daily dosing (97 % and 91.5 % respectively). The incidence of new thromboembolic events was statistically similar (3.5 % vs. 2 %, *p* = 0.72).

As noted previously, a subgroup analysis from a retrospective study suggested that VTE may recur more often in overweight and obese patients (BMI > 27 kg/m^2^) treated with enoxaparin once daily compared to twice daily (7.3 % vs. 3.4 %; OR 4.0 [CI 1.08–15]) [43]. This difference may reflect the benefits of a higher total daily dose with the twice daily regimen. A retrospective study of 193 patients weighing >90 kg treated with dalteparin 200 International units/kg total body weight for VTE revealed only 2 major bleeding events which were deemed unlikely to be caused by dalteparin [68].

There are limited data on dosing LMWH in patients with low body weight. The lowest body weight reported in an enoxaparin VTE clinical trial was 44 kg and patients <40 kg were excluded from the major dalteparin VTE clinical trial [43, 69]. A registry of 7962 patients receiving LMWH for acute VTE analyzed clinical outcomes based on weight ranges: (less than 50 kg vs. 50–100 kg, vs. greater than 100 kg] [17]. The majority of patients weighed between 50–100 kg; only 242 patients weighed >100 kg and only 161 patients weighed <50 kg. Compared to patients weighing 50–100 kg, patients <50 kg had a significant increase in the incidence of major bleeding (3 % vs. 1.3 %) and minor bleeding (5.3 % vs. 2.5 % [OR 2.2; 95 %CI 1.2–4]). Mean daily doses were significantly higher in the <50 kg group with 54 % receiving >200 international units/kg daily. The incidence of recurrent VTE was similar between the <50 kg and 50–100 kg groups. Patients >100 kg experienced similar bleeding and thromboembolic complications compared with the 50–100 kg group.

For patients weighting >190 kg, peak anti-Xa monitoring has been suggested [59]. However, an open label prospective trial in 233 patients showed that mean peak anti-Xa levels were similar between obese and healthy weight individuals receiving enoxaparin 1.5 mg/kg once daily or 1 mg/kg twice daily [70] and peak anti-Xa levels have not been correlated with effectiveness (see below).

#### **Guidance Statement**

*We suggest that in all patients, including underweight and obese, LMWH dosing should be based on total body weight. For patients* <*40* *kg, UFH may be more appropriate. For enoxaparin dosing in obese patients, 1* *mg/kg BID is preferred over 1.5* *mg/kg daily. Dose capping should be avoided. Routine monitoring of peak anti*-*Xa levels is not suggested in patients on LMWH, whether obese or non*-*obese.*

(3) How should patients with renal impairment be treated?

LMWHs are cleared renally. There is an inverse relationship between CrCl and anti-Xa levels, with accumulation of anti-Xa activity at the end of the dosing interval as renal function declines [12, 71, 72].

Enoxaparin appears to be more dependent on renal function for elimination than is dalteparin [73]. Product information for enoxaparin includes a dose reduction to 1 mg/kg daily for patients with CrCl < 30 mL/min [57]. A pharmacokinetic study showed that using this reduced dose of enoxaparin resulted in 74 % of peak anti-Xa levels being within an expected range of values [74]. After repeated dosing, higher peak anti-Xa levels were reported in patients receiving enoxaparin 1 mg/kg BID compared to 1.5 mg/kg daily in both moderate (CrCl 30–50 mL/min) and severe (CrCl < 30 mL/min) renal dysfunction, indicating greater accumulation with the BID regimen in renal insufficiency [70].

In comparison, dalteparin product information includes no dose adjustment for patients with severe renal impairment, and recommends to use with caution and “monitor anti-Xa levels” in patients with CrCl < 30 mL/min) [58]. One prospective study evaluating dalteparin 100 International units/kg every 12 h found no difference in peak anti-Xa levels in patients with CrCl < 40 mL/min compared to patients with normal renal function (0.47 U/mL vs. 0.55 U/mL, *p* > 0.5.) [75].

Compared to patients with normal renal function, the risk of major bleeding increases in patients with renal insufficiency exposed to LMWH. In a prospective registry of 1037 patients on LMWH, patients with CrCl < 30 mL/min had an increased incidence of major bleeding (7.3 % vs. 2.3 %; *p* < 0.001) [76]. A systematic review and meta-analysis of 18 LMWH studies (4971 patients) showed that patients with CrCl ≤ 30 mL/min had a significant increase in major bleeding compared to patients with CrCl > 30 mL/min (5 % vs. 2.4 %; odds ratio 2.25 [95 % CI, 1.19–4.27]; *p* = 0.013) [72]. Fifteen of the 18 studies evaluated enoxaparin and seven of those involved the use of therapeutic dosing rather than dosing for VTE prophylaxis. When data were analyzed based on LMWH preparation, major bleeding was increased with standard dose enoxaparin (8.3 % vs. 2.4 %; odds ratio, 3.88 [CI 1.78–8.45]) but not when dose was adjusted for CrCl (0.9 % vs. 1.9 %; odds ratio, 0.58 [CI, 0.09–3.78] *p* = 0.23). There were no data on bleeding associated with the use of dalteparin.

An increased risk of bleeding has also been observed in patients with moderate renal impairment. A retrospective study compared major bleeding in patients receiving treatment dose enoxaparin with normal renal function (CrCl > 80 mL/min) and moderate renal impairment (CrCl 30–50 mL/min) [77]. The incidence of major bleeding was 5.7 % with normal renal function compared to 22 % with moderate renal impairment, unadjusted odds ratio of 4.7 (95 % CI, 1.7–13; *p* = 0.002). A dose reduction for enoxaparin use in patients with mild or moderate renal impairment (CrCl 30–80 mL/min) has not been established.

Extended use (>10 days) of enoxaparin in patients with renal insufficiency may require trough anti-Xa measurement and dose adjustments if accumulation is noted. More data on dose adjustment in renal impairment are needed. One clinical approach to dosing enoxaparin in renal insufficiency is to utilize manufacture dose recommendations for CrCl < 30 mL/min but avoid LMWH if the CrCl < 20 mL/min [59].

LMWH is routinely avoided in patients on renal replacement therapy (RRT) because of the numerous variables that can affect clearance (filter type, interruption, regimen change). LMWH dose adjustments for RRT are not well defined [59].

#### **Guidance Statement**

*When LMWH is used for acute treatment of VTE in patients with renal impairment, we suggest that vigilant attention to potential bleeding risk and monitoring for signs and symptoms of bleeding be employed. Renal function should be estimated using the Cockcroft*-*Gault method for calculating CrCl. In patients with a CrCl* < *30* *mL/min the use of UFH may be preferred over LMWH and if enoxaparin is used, it should be dosed at 1* *mg/kg daily. If LMWH is used for an extended period beyond the usual 5*–*7* *days of treatment, trough anti*-*Xa measurement may be considered in patients with severe renal dysfunction. LMWH should be avoided in patients with CrCl* < *20* *mL/min and those receiving renal replacement therapy.*

(4) How should routine treatment be monitored?

LMWHs have predictable pharmacodynamic profiles and wide therapeutic windows that do not require routine coagulation monitoring in clinically stable and uncomplicated patients. There are currently no commercial assays available for LMWH. PT and aPTT are insensitive measures of LMWH activity. Anti-Xa activity is a surrogate marker that measures the anticoagulant effect of LMWH and is assumed to correlate with hemorrhagic and thromboembolic events. While LMWH anti-Xa concentrations may be helpful in evaluating dosing in special patient populations, routine LMWH anti-Xa monitoring is unnecessary and potentially harmful if misinterpreted [12, 59, 78].

Although the risk of HIT is < 1 % in patients on LMWH, the consequences of HIT can be devastating. Therefore, in patients with acute VTE, we suggest that a baseline platelet count be obtained prior to initiation of LMWH, and occasionally during the first 2 weeks of LMWH use. Circulating HIT antibodies may remain present for a median of 50–85 days depending on assay performed and re-exposure can lead to a large decrease in platelet count within 24 h. Therefore, in patients recently treated with heparin/LMWH, a baseline platelet count should be obtained prior to initiating LMWH and repeated 24 h later [63].

LMWHs are excreted by the kidney and accumulation may occur in renal impairment. Occasional monitoring of renal function using serum creatinine, and calculation of CrCl using the Cockcroft-Gault method may be useful to assess changes in renal function that may indicate the need for a dosing adjustment.

#### **Guidance Statement**

*We suggest all patients receiving LMWH be monitored for signs and symptoms of bleeding and be observed for changes in renal function that may require a dose adjustment. We suggest against the routine use of LMWH anti*-*Xa monitoring. CBC, platelet count and Scr should be assessed periodically during LMWH treatment.*

(5) Is there a role for peak anti-Xa monitoring and for trough anti-Xa monitoring?

The clinical trials evaluating LMWH did not use anti-Xa levels to guide dosing and anti-Xa levels have not been evaluated in large studies. Although anti-Xa levels have been used as a marker of LMWH activity they are not routinely evaluated in clinically stable or uncomplicated patients. The interpretation of anti-Xa levels depends on the dose and time of last LMWH administration. Trough anti-Xa levels may be used to evaluate accumulation of anticoagulant effect at the end of dosing interval. The value of peak anti-Xa levels is less clear. Peak levels occur 3–5 h after a LMWH dose and if obtained, should be measured at steady state [12, 79]. In a retrospective review, the majority of anti-Xa levels were drawn inappropriately, limiting their utility for interpretation [68].

Data supporting a relationship between elevated LMWH anti-Xa levels and bleeding are quite limited and include a study in which dalteparin was administered by continuous infusion and bleeding was increased in those with mean levels above 0.8 u/mL [80, 81]. In the uncommon situations in which anti-Xa activity is monitored, it should be determined using a chromogenic method and a calibration curve based on the LMWH used. Target anti-Xa levels are not clinically validated, and there is no standardized method for adjusting doses based on anti-Xa level [59]. Peak anti-Xa levels observed in patients treated with enoxaparin range from 0.6–1 IU/mL for twice daily dosing and >1 IU/mL for once daily dosing. For dalteparin, observed peaks may be somewhat higher simply because the total dose is given as a single injection (200 units/kg SQ once daily) rather than divided into two doses as in the case of enoxaparin (1 mg/kg SQ q12 h) [12, 79, 82]. Importantly, there are no data to suggest that making dosing adjustments based on peak levels is correlated with improved safety or efficacy.

While there is no consensus on an acceptable trough anti-Xa level for treatment dose LMWH, at the end of the 12 or 24 h dosing interval, these values should not be ‘high’ [83]. In an acute coronary syndrome study, trough anti-Xa levels >0.5 IU/mL were considered to be elevated [84]. Elevated troughs reflect lack of LMWH clearance and may suggest both an increased risk of bleeding and the need for a prolonged dosing interval.

Trough anti-Xa concentrations may be helpful to evaluate the safety of LMWH dosing in special patient populations including patients with severe renal impairment (although usefulness undetermined in patients on RRT) and extremely low body weight [12, 59]. The role of peak anti-Xa concentrations for evaluating efficacy in special populations including pregnancy and extremes of body weight is not defined.

#### **Guidance Statement**

*We suggest that in limited populations, including patients with severe renal failure, trough anti*-*Xa levels may have a role in evaluating LMWH accumulation and the need to prolong the dosing interval. We suggest that peak anti*-*Xa levels not be utilized to evaluate dosing regimens in clinical practice.*

(6) What is the appropriate duration of therapy when transitioning to oral anticoagulant therapy?

Traditional anticoagulation involves concurrent initiation of LMWH and warfarin on the same day, with continuation of LMWH for a minimum of 5 days and until the INR is above 2.0 (see above and Table 3) [11] When dabigatran or edoxaban are used for VTE treatment, LMWH must be started first and continued for a minimum of 5 days prior to initiation of these oral anticoagulants [47, 49, 50].

LMWH alone is an option for patients in whom INR is difficult to control or in whom oral anticoagulation is not an option, and is more effective than VKA therapy in patients with cancer [69]. Several randomized, controlled trials have evaluated the safety and efficacy of LMWH for the full course of VTE treatment compared to traditional short term LMWH followed by oral vitamin K antagonist (VKA) therapy. Among trials with the highest methodological quality, a recent meta-analysis showed a non-significant reduction in the odds of recurrent VTE (OR 0.80, 95 % CI 0.54–1.18) and in the odds of bleeding (OR 0.62, 95 % CI 0.36–1.07) favoring LMWH [85]. Nevertheless, oral anticoagulation remains a more common approach due to the expense of LMWH and the need for drug delivery by injection.

#### **Guidance Statement**

*Parenteral anticoagulation with LMWH should be overlapped with warfarin for at least 5* *days and until a single INR is 2.0 or greater. Treatment of VTE with rivaroxaban and apixaban does not require initial parenteral anticoagulation while dabigatran and edoxaban require a minimum of 5* *days of parenteral anticoagulation prior to initiation. See Table* *4**for additional details. The timing of the first dose of a TSOAC is based on when the next scheduled dose of LMWH would be due.*

(7) Which patients are acceptable candidates for outpatient treatment of VTE with LMWH?

A number of randomized trials have compared outpatient treatment of DVT with LMWH versus inpatient treatment of DVT with either UFH or LMWH. A Cochrane review of 6 randomized controlled trials including 1708 patients with DVT showed that outpatient therapy was associated with a lower rate of recurrent VTE, reduced mortality and no difference in minor bleeding [86].

While PE has historically been treated on an inpatient basis, a systematic review and meta-analysis of 11 studies, including 1258 patients, showed that low risk patients with PE can safety be treated as outpatients [87]. Studies included in the meta-analysis utilized either a risk stratification method or clinical judgment to determine low risk patients. The incidence of VTE recurrence and major bleeding was low in the studies and the event rates between the studies that used a risk stratification model versus clinical judgment were similar. Approximately one-third to one-half of acute PE patients may be classified as low-risk [88].

Clinical prediction rules, including the Pulmonary Embolism Severity Index (PESI) and Simplified PESI are simple tools to identify low-risk PE patients (mortality <1 %) with excellent negative predictive performance [89]. In a randomized controlled trial, acute PE patients with low risk PESI scores who were treated as outpatients with enoxaparin 1 mg/kg twice daily had non-inferior outcomes compared to those treated as inpatients [90].

Characteristics of patients who may be less suitable for outpatient VTE management have been identified and include body weight <70 kg, active malignancy, recent immobility, chronic heart failure, renal insufficiency, and bilateral DVT. In the REITE registry, these factors were independently associated with an increased risk of symptomatic PE, recurrent DVT, major bleeding or death [91]. In addition, DVT patients who may require hospitalization include those with venous gangrene or extensive iliofemoral involvement, severe acute obstruction (phlegmasia cerula dolens), poor social circumstances, active bleeding or a high risk of bleeding, severe pain, renal impairment, significant communication deficits or mobility problems [11, 92]. Home circumstances for adequate outpatient treatment include well-maintained living conditions, strong support from family or friends, phone access and ability to quickly return to hospital if there is clinical deterioration [11].

#### **Guidance Statement**

*We suggest patients with VTE be evaluated to determine treatment setting. Patients with DVT and/or PE who are identified as having a low risk of complications should be treated in the outpatient setting as long as they have adequate home support.*

(8) How should LMWH-induced over-anticoagulation, thrombocytopenia and bleeding be managed?

The overall risk of major bleeding associated with LMWH ranges from 1–4 % [93]. When significant bleeding or over-anticoagulation occurs, LMWH should be discontinued immediately. Observation without intervention is appropriate if bleeding is not present as demonstrated in a case series of intentional LMWH overdose [94]. Protamine sulfate may be used as a reversal agent for LMWH, but only reverses 60–80 % of the anticoagulant activity of LMWH. While it fully reverses the anti-IIa fraction of LMWH, it only partially reverses the anti-Xa component of LMWH due to the reduced sulfate charge in the ultra-low molecular weight heparin fragments present. Enoxaparin appears to be less susceptible to protamine sulfate reversal than dalteparin because its structure has less sulfonation [95].

There are limited clinical data on the use of protamine sulfate to reverse LMWH [93, 96]. A retrospective, single center study that evaluated the use of protamine sulfate to emergently reverse LMWH found that 4 of 14 patients with active bleeding continued to bleed or rebled after protamine administration [93]. No correlation was evident between anti-Xa levels and bleeding cessation.

The need for and dose of protamine sulfate is based on the timing of the last dose of LMWH, the severity of bleeding and the estimated clearance of LWMH based on the patient’s renal function. In patients with impaired renal function the anticoagulant effect of LMWH may persist and the treatment window for protamine sulfate may be extended. Current guidelines suggest that if LMWH was given within the previous 8 h, protamine sulfate may be administered in a dose of 1 mg per 100 anti-Xa units of LMWH up to a maximum single dose of 50 mg (1 mg of enoxaparin equals approximately 100 anti-Xa units) [12]. A second dose of 0.5 mg protamine sulfate per 100 anti-Xa units should be administered if bleeding continues or if the aPTT is prolonged 2–4 h after the initial protamine dose. A lower initial dose of protamine sulfate (0.5 mg per 100 anti-Xa units) may be administered if the time since LMWH administration is greater than 8 h. If greater than 12 h has elapsed since administration of LMWH, protamine sulfate may not be effective and supportive measures to control bleeding should be used.

The risk of HIT is lower for LMWH than for UFH [55]. Nevertheless, if thrombocytopenia or thrombosis develops during LMWH treatment, the patient should be evaluated for HIT as outlined previously for UFH, and treated according to current guidelines [55].

#### **Guidance Statement**

*We suggest that protamine sulfate be used to reverse LWMH if major bleeding occurs. The timing of the last dose of LMWH should be assessed when determining if protamine sulfate should be administered and the appropriate dose to be administered. A repeat dose of protamine may be administered if bleeding continues or if the aPTT is prolonged 2–4 h after the initial dose. Patients who develop HIT in response to LMWH should be treated according to current guidelines for HIT.*

## Guidance

### Fondaparinux for the treatment of acute VTE

1. How should fondaparinux be initiated, including baseline labs and dosing?

The clinical evidence supporting the efficacy and safety of fondaparinux in the treatment of VTE comes from the MATISSE trials. In the MATISSE-DVT trial, patients (n = 2205) with acute symptomatic DVT were randomized in a double-blinded fashion to SC fondaparinux or SC enoxaparin overlapping with a VKA for at least 5 days and until an INR of greater than 2.0 was achieved [97]. Fondaparinux was dosed based on weight terciles. Patients weighing 50–100 kg received 7.5 mg once daily, while patients weighing less than 50 kg received 5 mg once daily, and those weighing more than 100 kg received 10 mg once daily. Enoxaparin was dosed at 1 mg/kg twice daily.

In the MATISSE-PE trial, patients (n = 2213) with acute symptomatic PE were randomized in an open-label fashion to SC fondaparinux as dosed in the MATISSE-DVT trial, or intravenous (IV) UFH with an initial bolus of at least 5000 units and an initial infusion of at least 1250 units per hour to achieve and maintain an aPTT of 1.5–2.5 times control [98]. Both parenteral agents were to be overlapped with a VKA for at least 5 days and until an INR of greater than 2.0 was achieved.

In the MATISSE-DVT trial, fondaparinux was non-inferior to enoxaparin with respect to the primary endpoint of recurrent VTE at 3 months (3.9 % vs. 4.1 %) [97]. Major bleeding was also not different between fondaparinux and enoxaparin (1.1 % vs. 1.2 %). In the MATISSE-PE trial, fondaparinux was found to be non-inferior compared to IV UFH in preventing the primary endpoint of symptomatic PE and new or recurrent DVT at 3 months (3.8 % vs. 5.0 %) [98]. Major bleeding was also similar between the groups (1.3 % vs. 1.1 %). In both studies, mortality did not differ between the groups.

Therapy should be initiated as soon as possible and it is determined that fibrinolytics are not going to be administered. A baseline weight is required to determine the correct dose of fondaparinux. A baseline SCr and CrCl should be calculated since fondaparinux is contraindicated in patients with a CrCl of less than 30 mL/min. A baseline CBC should also be evaluated. An elevated pre-treatment PT or aPTT may detect the presence of an underlying coagulation defect.

#### **Guidance Statement**

*We suggest that total body weight, baseline serum creatinine, CBC, PT and aPTT be obtained prior to initiating fondaparinux therapy. We suggest dosing fondaparinux based on weight as follows: less than 50* *kg: 5* *mg SC once daily, 50–100* *kg: 7.5* *mg SC once daily, and greater than 100* *mg: 10* *mg SC once daily.*

(2) What weight should be used to calculate dosing, and should obese and low body weight patients be treated differently?

In the MATISSE trials, total body weight was used to calculate dosing of fondaparinux [97, 98]. In the combined MATISSE trials, 496 (11 %) of patients enrolled weighed more than 100 kg, and 251 of them received fondaparinux [99]. In patients weighing 100 kg or less, the incidence of recurrent VTE occurred in 3.9 % of patients receiving fondaparinux compared to 4.4 % with heparin (*p* = 0.42). In patients weighing more than 100 kg, the rate of recurrent VTE in patients receiving fondaparinux 10 mg SC once daily was 4.0 % compared to 5.7 % in patients receiving a heparin (*p* = 0.41). Major bleeding events occurred numerically less frequently in patients receiving fondaparinux 10 mg compared to other doses (0.4 % vs. 1.3 %) and was not different compared to patients weighing more than 100 kg receiving a heparin (0.4 % vs. 0.8 %; *p* = 0.62). A similar analysis was conducted in the 1216 patients (28 %) in the MATISSE trials with a BMI of 30 or more [99]. The rate of recurrent VTE was non-inferior with fondaparinux compared with heparin in both non-obese (3.9 % vs. 4.5 %; *p* = 0.42) and obese (3.7 % vs. 4.8 %; *p* = 0.40) patients. As with body weight, the efficacy of fondaparinux appeared to be similar regardless of BMI. Major bleeding rates were also not different between patients receiving fondaparinux and a heparin in non-obese (1.5 % vs. 1.2 %; *p* = 0.53) and obese (0.3 % vs. 1.1 %; *p* = 0.18) patients. The median weight in those over 100 kg in the MATISSE trials was 110 kg, with the heaviest patient weighing 175.5 kg. The median BMI in those with a BMI of 30 or more was 33 kg/m2, with the highest being 80.3 kg/m2. Based on these data, there does not seem to be any reason to treat heavier or obese patients with doses greater than 10 mg.

Data on low body weight patients are more limited. There were only a total of 102 (2.3 %) patients in the combined MATISSE trials with a body weight of less than 50 kg [97, 98]. The rate of recurrent VTE in the fondaparinux and heparin groups were similar (11.3 % vs. 14.3 %), but about 3-fold higher than the rates in the overall study (about 4 %). At first glance it may be interpreted that 5 mg may have been an insufficient dose, but rates were similar in the heparin arms. It should also be noted that the numbers are quite small (6 events for fondaparinux vs. 7 events for a heparin) and should be interpreted with caution. Major bleeding rates were consistently low for both the fondaparinux and heparin group (1.9 % vs. 2.0 %).

#### **Guidance Statement**

*We suggest patients be dosed based on total body weight. Patients weighing more than 100* *kg should receive fondaparinux 10* *mg SC once daily. Patients with a BMI greater than 30* *kg/m*^*2*^*should be dosed based on total body weight. Patients weighing less than 50* *kg should receive 5* *mg SC once daily.*

(3) How should patients with renal impairment be treated?

Fondaparinux is eliminated almost completely through the kidney as unchanged drug. Patients with a Scr greater than 2.0 mg/dL (177 μmol/L) were not included in the MATISSE trials [97, 98]. There were 51 (2.3 %) patients randomized to fondaparinux with a CrCl less than 30 mL/min enrolled in the MATISSE trials. In these patients, there were 4 major bleeding events (7.8 %) [97, 98].

Fondaparinux clearance is reduced by 25 % in patients with moderate renal insufficiency (CrCl 30–60 mL/min) [100]. Therefore, there could be some drug accumulation in these patients with longer than usual courses of therapy. It may be appropriate to monitor a trough fondaparinux anti-Xa to assess accumulation in patients receiving treatment dose for more than 10 days. While some lower dose trials have been conducted in the area of VTE prophylaxis in patients with a CrCl of 20–50 mL/min [100, 101] fondaparinux should be avoided in patients with a CrCl of less than 30 mL/min for treatment of VTE until more data become available. According to the manufacturer, fondaparinux is contraindicated in patients with a CrCl of less than 30 mL/min.

#### **Guidance Statement**

*We suggest that fondaparinux be avoided in patients with a CrCl of less than 30* *mL/min. We also suggest that patients with moderate renal insufficiency be monitored closely for bleeding during longer durations of therapy due to potential accumulation.*

(4) How should treatment be monitored?

Due to the predictable pharmacokinetic and pharmacodynamic profile of fondaparinux, routine coagulation monitoring is not necessary. As with any anticoagulant, the most common adverse effect is bleeding. Therefore, monitoring for signs and symptoms of bleeding is paramount. The need for platelet count monitoring is uncertain.

Fondaparinux has a limited impact on the PT or aPTT, even at high doses [102]. The mean increase in the PT at therapeutic levels of fondaparinux (0.8 μg/mL) was only about 1.2 s. Even at supratherapeutic levels (2 μg/mL) the PT only increased 1.8 s above baseline. The mean increase in the aPTT was only 4.6 s at therapeutic levels of fondaparinux, and 6.2 s with supratherapeutic levels. Therefore, these assays are not appropriate for measuring fondaparinux therapy.

#### **Guidance Statement**

*We suggest that most patients receiving fondaparinux do not require therapeutic drug monitoring. If the clinical setting suggests the need to assess accumulation of fondaparinux, we suggest using an anti*-*Xa assay calibrated for fondaparinux, and we suggest against the use of a PT, aPTT, or activated clotting time (ACT).*

(5) Is there a role for peak anti-Xa monitoring and for trough anti-Xa monitoring?

Most patients receiving fondaparinux should not receive anti-Xa monitoring. While fondaparinux provides a predictable anticoagulant response, there may be special situations in which measuring plasma concentrations may be helpful. While evidence to support measuring plasma concentrations is lacking, concentrations may be helpful to guide therapy in patients on long-term therapy, or with sudden changes in renal function, extremes in body weight, or pregnancy. However, there are no data to suggest that dosing adjustments in response to known plasma concentrations have any influence on patient outcomes.

A chromogenic anti Xa assay calibrated with fondaparinux produces reliable and reproducible results [102–105]. Fondaparinux needs to be used to form the standard curves to measure fondaparinux levels [12]. Results obtained using a LMWH standard curve are less accurate and standard curves using UFH are completely inaccurate and should not be used [102, 106]. Peak anti-Xa levels are typically achieved in 3 h after dosing [107]. Target anti-Xa levels for fondaparinux are not established; however peak anti-Xa levels in patients receiving treatment doses of fondaparinux range from 0.6 to 1.5 μg/mL and are typically in the range of 1.20–1.26 μg/mL. Observed trough levels in patients receiving fondaparinux are in the range of 0.46–0.62 μg/mL [12, 104, 108]. Importantly, these are observed values, not a “target range” and have not been correlated with clinical outcomes.

#### **Guidance Statement**

*If the clinical setting suggests a benefit of measuring trough fondaparinux levels, we suggest a chromogenic anti*-*Xa with the standardization curve calibrated with fondaparinux. We suggest against using anti*-*Xa with the standard curves created using a LMWH or UFH.*

(6) What is the appropriate duration of therapy when transitioning to oral anticoagulant therapy?

The duration of fondaparinux therapy when transitioning to a VKA is similar to that with UFH or a LMWH (Table 3). In the MATISSE trials, fondaparinux was overlapped with a VKA for at least 5 days and until an INR of greater than 2.0 was achieved. The mean duration of overlap was 7 days [97, 98].

If fondaparinux is administered to a patient who will be transitioned to a TSOAC, the oral agent should be initiated at the time that the next fondaparinux dose would have been given. See above and Table 4 for additional details.

#### **Guidance Statement**

*Parenteral anticoagulation with fondaparinux should be overlapped with warfarin for at least 5* *days and until a single INR is 2.0 or greater. Treatment of VTE with rivaroxaban and apixaban does not require initial parenteral anticoagulation while dabigatran and edoxaban require a minimum of 5* *days of parenteral anticoagulation prior to initiation. See Table* *4**for additional details. The timing of the first dose of a TSOAC is based on when the next scheduled dose of fondaparinux would be due.*

(7) Who is a candidate for outpatient treatment of VTE with fondaparinux?

In the MATISSE-DVT trial, 88 patients (8.1 %) were treated with fondaparinux completely on an out-patient basis, and 253 patients (23.2 %) received at least 3 days of outpatient therapy. These numbers were similar to the enoxaparin patients treated as outpatients (8.3 % and 25.2 %, respectively) [10]. In patients receiving some outpatient therapy, the rate of recurrent VTE was similar between the fondaparinux and enoxaparin groups (2.0 % vs. 4.3 %) and was similar to the event rates for the overall study. Major bleeding was also similar between groups (1.5 % vs. 0.8 %) and similar to the bleeding rates in the overall study. In the MATISSE-PE trial, 158 (14.5 %) of patients randomized to fondaparinux received some portion of their fondaparinux treatment on an outpatient basis [98]. The rate of recurrent VTE was 3.2 % and there were no major bleeding events in these patients. These values are similar to those in the overall study.

Outpatient treatment with fondaparinux has demonstrated similar efficacy and safety to inpatient treatment of VTE. Therefore, patients who would be considered outpatient candidates for LMWH therapy should also be considered outpatient candidates for fondaparinux.

#### **Guidance Statement**

*We suggest patients with VTE be evaluated to determine the treatment setting. Patients with DVT and/or PE who are identified as having a low risk of complications (see above) should be treated in the outpatient setting as long as they have adequate home circumstances.*

(8) Can fondaparinux be used for VTE treatment in the presence of active HIT or those with a history of HIT?

Due to the smaller size of the molecule and low affinity for platelet factor 4, fondaparinux does not cross react with HIT antibodies [109]. While there have been a small number of case reports of fondaparinux-associated HIT with the use of fondaparinux for VTE prevention [110–112], no cases of HIT have been reported in any of the major clinical trials evaluating the efficacy and safety of fondaparinux in the prevention of VTE, the treatment of VTE, or in treatment of patients with acute coronary syndrome. More data exist for the ability of fondaparinux to be used in the treatment of HIT or as a safe alternative in those with a history of HIT [113]. A number of case reports and case series support the potential role of using fondaparinux in the treatment of HIT [114–116]. These reports comprise over 70 patients who developed HIT after treatment with UFH and/or a LMWH for VTE prophylaxis. Fondaparinux doses varied from 2.5 to 15 mg daily.

In the only prospective study in the literature, 7 patients with acute HIT were treated with fondaparinux and compared to 10 similar historical control HIT patients from the same hospital [117]. Patients presenting with thrombosis (6 of the 7) received treatment doses of fondaparinux based on weight as in the MATISSE trials, while the patient presenting without thrombosis received 2.5 mg of fondaparinux SC once daily. Historical controls received an injectable direct thrombin inhibitor via the hospital’s protocol. Eight of the 10 historical control patients presented with thrombosis. All fondaparinux patients experienced platelet count recovery compared to 8 of the 10 historical controls. There were no new thromboses, major bleeding events, or death in the fondaparinux treated patients. There were 2 deaths in the historical control patient group.

One benefit of fondaparinux over direct thrombin inhibitors for treating HIT is that clinicians do not need to be concerned with PTT confounding which is defined as “a situation where an underlying patient-related clinical factor (or factors) results in anticoagulant related changes in PTT values that are misleading with respect to indicating a patient’s true level of anticoagulation [118]. PTT confounding can be caused by disseminated intravascular coagulation, hepatic failure, VKA use and lupus anticoagulant. With PTT confounding, patients on DTIs appear supratherapeutic with high aPTTs and as a result DTI therapy is held or reduced. Progressive thrombosis then ensues while the aPTT remains elevated. Although fondaparinux may offer a benefit, its long elimination half-life, dependence on renal function for elimination and non-reversibility must be considered.

In the most recent guidelines from ACCP, fondaparinux is mentioned as an option for the treatment of patients with HIT without thrombosis, as well as for use in patients with a history of HIT who require anticoagulation [63]. While the guidelines mention the limitations of the data, it should be remembered that there are no high quality data available for any agent in the treatment of HIT.

#### **Guidance Statement**

*We suggest that fondaparinux may be used for treatment of VTE in patients with a history of HIT. In patients with acute VTE who develop HIT in response to initial use of UFH/LMWH, we suggest that fondaparinux may be considered as an option for treatment. When fondaparinux is used in patients with acute HIT, we suggest that treatment doses be used.*

(9) How should fondaparinux-induced over-anticoagulation and bleeding be managed?

While protamine sulfate is effective at reversing the anticoagulant effect of UFH and to some extent LMWH, it is not effective in reversing fondaparinux [119]. Since protamine does not bind to the low molecular weight molecule due to the reduced sulfate charge of fondaparinux, there is no reversal of the anticoagulant effect. Recombinant activated factor VII (rFVIIa) is the most extensively studied reversal agent for fondaparinux [120]. Healthy subjects who received a single 10 mg SC dose of fondaparinux followed by a single IV bolus of rFVIIa 90 µg/kg 2 h later demonstrated normalization of thrombin-generation time and endogenous thrombin potential for up to 6 h. Case reports and case series also support a role for rFVIIa in stopping fondaparinux-induced bleeding [121–123]. Available data support the use of high dose rFVIIa at 90 µg/kg. The efficacy of a lower and less expensive dose of 30 µg/kg is unknown. The ability of concentrated clotting factors to reverse the impact of fondaparinux on thrombin generation test has been evaluated in one in vitro study [124]. In this study, activated prothrombin complex concentrate (FEIBA-NF^®^) was able to correct the thrombin generation test, while prothrombin complex concentrate (Kaskadil^®^) and rFVIIa were less effective. More data and experience with these agents are needed, particularly considering the risk of thrombosis associated with their use.

#### **Guidance Statement**

*We suggest the use of rFVIIa in the setting of life threatening bleeding induced by fondaparinux, and that potential benefits must be weighed against thrombotic risk.*

## Conclusion

Despite advances in the development of oral anticoagulants, the parenteral heparins continue to be a component of the treatment of VTE. Their appropriate use, particularly in special populations, remains a challenge for clinicians. Table 5 summarizes the guidance statements for the practical management of the heparin anticoagulants.

**Table 5:** Summary of guidance statements

Question	Guidance statement
Heparin for treatment of acute VTE	
(1) How should heparin be initiated, including baseline laboratory tests and dosing?	We suggest that total body weight, CBC, PT and aPTT be obtained prior to initiating heparin therapy. Heparin efficacy is related to dose regardless of route. The initial dose is more important than the aPTT in predicting efficacy. Although optimal initial dosing for bolus and continuous infusion remain uncertain, we suggest doses outlined in Table 2, acknowledging that these options have not been compared in head-to-head clinical trials. Internal audits to determine the dose requirement to produce therapeutic anticoagulation based upon the responsiveness of the health-system’s aPTT reagent and coagulation instrument are encouraged
(2) What weight should be used to calculate dosing, and should obese and low body weight patients be treated differently?	When a weight based heparin dosing strategy is selected, we suggest total body weight for calculating dose and encourage internal audits of protocol performance. For the obese/morbidly obese patient either total body weight or adjusted body weight can be used. Although no increased risk of major bleeding has been reported when morbidly obese patients are managed using total body weight, studies have not included patients weighing above 270 kg. If adjusted body weight is used, prompt attention to initial laboratory results is warranted to ensure the therapeutic threshold is exceeded in a timely manner. Empiric dose caps may increase the risk of initial under anticoagulation in obese and morbidly obese patients. If empiric dose caps are used, individualized initial dosing should be available for obese and morbidly obese patients
(3) How should heparin be monitored?	The optimal approach to heparin monitoring is unknown. Either aPTT or heparin anti-Xa level monitoring may be used. We suggest using anti-Xa level monitoring in patients with heparin resistance, a prolonged baseline aPTT or altered heparin responsiveness. We suggest the aPTT or anti-Xa level be checked every 6 h until two consecutive therapeutic results are obtained, after which the frequency of monitoring can be extended to once daily
(4) What data support the benefit of monitoring?	The benefit of monitoring IV heparin once a therapeutic threshold has been exceeded is not well defined. We suggest monitoring of continuous infusion heparin therapy, either using aPTT or anti-Xa, as this is considered standard of care despite the weak evidence base. Monitoring is optional in those receiving SC weight-based heparin therapy
(5) What is the appropriate therapeutic range?	The optimal heparin therapeutic range is uncertain. The target therapeutic range is less important than ensuring an appropriate initial heparin dose. The CAP recommends the one-time establishment of a heparin concentration-derived aPTT therapeutic range. The cumulative summation method is suggested for range re-evaluation following reagent/instrument change. When anti-Xa monitoring is used, a therapeutic target of 0.3–0.7 units/mL is suggested
(6) When should heparin resistance be suspected?	We suggest drawing a paired aPTT and heparin anti-Xa level when heparin resistance is suspected. If the aPTT is subtherapeutic and the anti-Xa level is therapeutic, the heparin dose does not require adjustment and subsequent monitoring should occur using the anti-Xa level when feasible. If, despite serial dose increases, both the aPTT and anti-Xa level remain low, true heparin resistance may be present
(7) What algorithm should be used for dosing adjustments?	We recommend that heparin dosing be guided by a dose adjustment nomogram, and that a weight based heparin dose adjustment algorithm may offer benefit over a fixed adjustment algorithm for the obese patient. More research in defining and assessing the optimal dosage adjustment nomogram is needed
(8) What is the appropriate duration of therapy for heparin for transition to oral anticoagulant therapy?	Parenteral anticoagulation with heparin should be overlapped with warfarin for at least 5 days and until a single INR is 2.0 or greater. Treatment of VTE with rivaroxaban and apixaban does not require initial parenteral anticoagulation while dabigatran and edoxaban requires a minimum of 5 days of parenteral anticoagulation prior to initiation. See Table 4 for additional details
(9) How should heparin-induced over-anticoagulation, thrombocytopenia and bleeding be managed?	We suggest protamine sulfate be administered to reverse the effect of heparin when indicated. We suggest that health systems develop and implement guidelines on anticoagulant reversal and HIT evaluation and management
LMWH for treatment of acute VTE	
(1) How should LMWH be initiated, including baseline laboratory tests and dosing?	We suggest that total body weight, baseline serum creatinine, CBC, PT and aPTT be obtained prior to initiating LMWH. We suggest that when enoxaparin is used for the treatment of VTE, only the twice daily dosing strategy be used, except in patients with severe renal insufficiency (see below). Further, we suggest that once daily dalteparin can be used for the treatment of both cancer- and non-cancer-associated VTE
(2) What weight should be used to calculate dosing, and should obese and low body weight patients be treated differently?	We suggest that in all patients, including underweight and obese, LMWH dosing should be based on total body weight. For patients <40 kg, UFH may be more appropriate. For enoxaparin dosing in obese patients, 1 mg/kg BID is preferred over 1.5 mg/kg daily. Dose capping should be avoided. Routine monitoring of peak anti-Xa levels is not recommended in patients on LMWH, whether obese or non-obese
(3) How should patients with renal impairment be treated?	When LMWH is used for acute treatment of VTE in patients with renal impairment, we suggest that vigilant attention to potential bleeding risk and monitoring for signs and symptoms of bleeding be employed. Renal function should be estimated using the Cockcroft-Gault method for calculating CrCl. In patients with a CrCl < 30 mL/min the use of UFH may be preferred over LMWH and if enoxaparin is used, it should be dosed at 1 mg/kg daily. If LMWH is used for an extended period beyond the usual 5–7 days of treatment, trough anti-Xa measurement may be considered in patients with severe renal dysfunction. LMWH should be avoided in patients with CrCl < 20 mL/min and those receiving renal replacement therapy
(4) How should routine treatment be monitored?	We suggest all patients receiving LMWH be monitored for signs and symptoms of bleeding and be observed for changes in renal function that may require a dose adjustment. We suggest against the routine use of LMWH anti-Xa monitoring. CBC, platelet count and Scr should be assessed periodically during LMWH treatment
(5) Is there a role for peak anti-Xa monitoring and for trough anti-Xa monitoring?	We suggest that in limited populations, including patients with severe renal failure, trough anti-Xa levels may have a role in evaluating LMWH accumulation and the need to prolong the dosing interval. We suggest that peak anti-Xa levels not be utilized to evaluate dosing regimens in clinical practice
(6) What is the appropriate duration of therapy when transitioning to oral anticoagulant therapy?	Parenteral anticoagulation with LMWH should be overlapped with warfarin for at least 5 days and until a single INR is 2.0 or greater. Treatment of VTE with rivaroxaban and apixaban does not require initial parenteral anticoagulation while dabigatran and exoxaban require a minimum of 5 days of parenteral anticoagulation prior to initiation. See Table 4 for additional details. The timing of the first dose of a TSOAC is based on when the next scheduled dose of LMWH would be due
(7) Which patients are acceptable candidates for outpatient treatment of VTE with LMWH?	We suggest patients with VTE be evaluated to determine treatment setting. Patients with DVT and/or PE who are identified as having a low risk of complications should be treated in the outpatient setting as long as they have adequate home circumstances
(8) How should LMWH-induced over-anticoagulation, thrombocytopenia and bleeding be managed?	We suggest that protamine sulfate be used to reverse LWMH if major bleeding occurs. The timing of the last dose of LMWH should be assessed when determining if protamine sulfate should be administered and the appropriate dose to be administered. A repeat dose of protamine may be administered if bleeding continues or if the aPTT is prolonged 2–4 h after the initial dose. Patients who develop HIT in response to LMWH should be treated according to current guidelines for HIT
Fondaparinux for treatment of acute VTE	
(1) How should fondaparinux be initiated, including baseline labs and dosing?	We suggest that total body weight, baseline serum creatinine, CBC, PT and aPTT be obtained prior to initiating fondaparinux therapy. We suggest dosing fondaparinux based on weight as follows: less than 50 kg: 5 mg SC once daily, 50–100 kg: 7.5 mg SC once daily, and greater than 100 mg: 10 mg SC once daily
(2) What weight should be used to calculate dosing, and should obese and low body weight patients be treated differently?	We suggest patients be dosed based on total body weight. Patients weighing more than 100 kg should receive fondaparinux 10 mg SC once daily. Patients with a BMI greater than 30 kg/m2 should be dosed based on total body weight. Patients weighing less than 50 kg should receive 5 mg SC once daily
(3) How should patients with renal impairment be treated?	We suggest that fondaparinux be avoided in patients with a CrCl of less than 30 mL/min. We also suggest that patients with moderate renal insufficiency be monitored closely for bleeding during longer durations of therapy due to potential accumulation
(4) How should treatment be monitored?	We suggest that most patients receiving fondaparinux do not require therapeutic drug monitoring. If the clinical setting suggests the need to assess accumulation of fondaparinux, we suggest using an anti-Xa assay calibrated for fondaparinux, and we suggest against the use of a PT, aPTT, or ACT
(5) Is there a role for peak anti-Xa monitoring and for trough anti-Xa monitoring?	If the clinical setting suggests a benefit of measuring trough fondaparinux levels, we suggest a chromogenic anti-Xa with the standardization curve calibrated with fondaparinux. We suggest against using anti-Xa with the standard curves created using a LMWH or UFH
(6) What is the appropriate duration of therapy when transitioning to oral anticoagulant therapy?	Parenteral anticoagulation with fondaparinux should be overlapped with warfarin for at least 5 days and until a single INR is 2.0 or greater. Treatment of VTE with rivaroxaban and apixaban does not require initial parenteral anticoagulation while dabigatran and edoxaban require a minimum of 5 days of parenteral anticoagulation prior to initiation. See Table 4 for additional details. The timing of the first dose of a TSOAC is based on when the next scheduled dose of fondaparinux would be due
(7) Who is a candidate for outpatient treatment of VTE with fondaparinux?	We suggest patients with VTE be evaluated to determine the treatment setting. Patients with DVT and/or PE who are identified as having a low risk of complications (see above) should be treated in the outpatient setting as long as they have adequate home circumstances
(8) Can fondaparinux be used for VTE treatment in the presence of active heparin-induced thrombocytopenia (HIT) or those with a history of HIT?	We suggest that fondaparinux may be used for treatment of VTE in patients with a history of HIT. In patients with acute VTE who develop HIT in response to initial use of UFH/LMWH, we suggest that fondaparinux may be considered as an option for treatment. When fondaparinux is used in patients with acute HIT, we suggest that treatment doses be used
(9) How should fondaparinux-induced over-anticoagulation and bleeding be managed?	We suggest the use of rFVIIa in the setting of life-threatening bleeding induced by fondaparinux, and that potential benefits must be weighed against thrombotic risk
